# Salty Crackers as Fortuitous Dosimeters: A Novel PSL Method for Rapid Radiation Triage

**DOI:** 10.3389/fpubh.2021.661376

**Published:** 2021-04-09

**Authors:** Nadica Maltar-Strmečki, Monica Vidotto, Sara Della Monaca, Ina Erceg, Paola Fattibene, Maja Vojnić Kortmiš, Maria Cristina Quattrini, Emanuela Bortolin

**Affiliations:** ^1^Division of Physical Chemistry, Ruder Bošković Institute (RBI), Zagreb, Croatia; ^2^Core Facilities, Istituto Superiore di Sanità (ISS), Roma, Italy

**Keywords:** retrospective dosimetry, fortuitous dosimeters, photo-stimulated luminescence, radiation triage, salty snacks

## Abstract

When a radiological and nuclear (R/N) emergency occurs, the categorization of individuals into those who are unaffected and those requiring medical intervention is a high priority. At times, a professional dosimeter is not available and therefore some common belongings may be used as fortuitous dosimeters. The preparation of these objects for the measurement should be such as to give the most accurate and precise results. This paper focused on the Photo-Stimulated Luminescence (PSL) response of salty crackers confronts the problem of sample preparation (mass, grain size), dose response and signal stability. The dose response was determined for doses up to 5 Gy, which allowed the calculation of the limit of detection. Additionally, the signal stability was investigated for samples irradiated with 0.3 and 3 Gy. The observed decrease of the signal does not prevent the detection in the dose range typical for R/N emergency. The main dosimetric characteristics were investigated by using two different models of PSL readers equipped with single (infrared) or double (infrared, blue light) stimulation. The results indicated that the limit of detection can be improved by applying blue light stimulation. Moreover, strong correlation of the measurements performed in the two different instruments, as well as the rapidity of the analysis and the simplicity of the operations, suggest that this method can be suitable for a rapid radiation triage of a large number of civilians in a mass casualty event. The study was simultaneously conducted by two laboratories (Ruder Bošković Institute, RBI, Croatia and Istituto Superiore di Sanità, ISS, Italy) involved in the *BioPhyMeTRE* project (grant No. G5684) supported by NATO Science for Peace and Security Programme.

## Introduction

Retrospective dosimetry plays a key role in the determination of the absorbed dose due to an accident in a nuclear reactor, a terrorist attack, a nuclear weapon explosion or a small-scale accident that could occur in medicine or industry (1–3). In the absence of a professional dosimeter, some common objects belonging to the individuals involved in the accident could serve as fortuitous dosimeters. Those are analyzed by different physical techniques. The stimulated luminescence techniques, in particular, have been successfully applied to various materials taken from personal objects like watches, electronic devices, ID cards, cigarettes, banknotes, paper tissues, mobile phone parts, salty snacks, etc. (4–9). Of particular interest are the results obtained with objects of no considerable value which people would easily donate in an emergency situation. Starting from these findings, a novel method, based on the use of a low-cost portable instrument for Photo-Stimulated Luminescence (PSL) have been tested on above mentioned inexpensive matrices. This type of PSL reader was primarily designed to identify irradiated food by detecting the luminescence emitted, after infrared stimulation, by the silicates contaminating the foodstuffs but, in principle, it could be applied for different purposes on other materials with luminescence properties. Different models/brands are available around the world and used mainly for food control; their characteristics are small size and weight which make them portable, low cost and simple for application on the scene of the accident. New models are also available, additionally offering double stimulation by infrared and visible radiation which makes them more suitable for the application in retrospective dosimetry. These PSL systems allow to perform measurements directly on the materials without any pre-treatment, thus making the analyses extremely rapid. All these aspects/advantages allow to propose the method for a fast triage of a large number of potential victims which would considerably improve response speed, quality, cost and effectiveness of medical care in general. Fast triage of a large group of people is needed to separate individuals needing specific treatment followed by timely medical decisions according to the dosimetric triage categorization. The individuals should be divided in three different categories (low: <1 Gy, medium: 1–2 Gy, and high: >2 Gy). Therefore, chosen dose range is related to the doses of interest for emergency triage, which are >0.5 Gy (1, 2). Particular attention was given to 1 Gy limit which divides individuals in those needing medical care and those who are mostly unaffected not requiring immediate care.

This paper reports the results of a PSL study carried out on a popular brand of salty crackers which are very popular among children and adolescents worldwide. The consumption of salt-containing snacks is increasing (10) and we can expect to find them on the location of the accident. In this work the main dosimetric characteristics, i.e., dose response, detection limit and signal stability, were investigated by using two different models of PSL readers equipped with single (infrared) or double (infrared and blue light) stimulation. Particular attention was paid to the sample preparation procedure, of crucial importance for the reproducibility of the data.

## Materials and Methods

TUC crackers (Original) were purchased at a local store or vending machines for snacks and drinks. Declared salt content of all samples is 1.7 g/100 g independently of the country were distributed. The allowed salt content uncertainty is ±0.3 g/100 g according to EU Regulation (11). All samples were packed in the characteristic yellow non-transparent polypropylene bag.

Irradiation was carried out with Cobalt-60 in a calibration teletherapy unit Co-60 Alcyon, CIS Bio International, available at RBI and with the Cs-137 source of the Gammacell 40 facility available at ISS with a dose rate of about 3.26 Gy/min and 0.7 Gy/min, respectively.

Sample preparation and sample storage were carried out at room temperature in dry air, and to avoid the bleaching of PSL signal in absence of light. The crackers were crumbled with a pestle, weighted and placed in Petri dishes (50 mm in diameter). Measurements were carried out with different models of SUERC PSL readers (Scotland/UK): a portable OSL Reader V.2.4 with three types of stimulation: infrared (890 nm), blue light (470 nm) and both simultaneously combined and an Irradiated Food Screening System with infrared stimulation (890 nm). Further in the text, the first one will be referred to as Reader 1, while the second one will be referred to as Reader 2. Data was acquired for 60 s for each sample and is expressed in “total counts”—an arbitrary unit of the instrument.

Measurements regarding the dose response within the range 0.1 to 5.0 Gy were performed within 1 h after the irradiation process; those obtained for the determination of the effects of sample mass (1 g and 2 g with an error of ±1%) and grain size (bigger ~1 cm, medium ~0.5 cm, completely crumbled <0.1 cm) were done 24 h after irradiation, while those meant to determine the signal stability took place at different times after irradiation ranging from 1 h to 20 days. Each different set of measurements was repeated 6 times. The data was analyzed using the software SigmaPlot V13 (Systat Software GmbH).

## Results and Discussion

### Effects of Mass and Grain Size

One of the main goals of this study was to determine the most suitable method to prepare the cracker samples for the potential application. The cracker's component that gives the PSL signal are the defects in the salt crystals (7–9) that seem to be mostly present on the surface of the cracker. This opens the possibility of dealing with a non-homogeneous sample that would prevent a proper assessment of the dose absorbed by the individual involved in the R/N accident.

Tables 1, 2 represent the response with the corresponding relative standard deviations for samples of masses 1 g and 2 g, for the unirradiated and irradiated with 1 Gy, respectively. Besides sample mass (samples weighting 1 g or 2 g) and the size of the cracker grains that are meant to be placed in the Petri dish (three different grain sizes), three different stimulations, blue light (470 nm), infrared (890 nm) and combined using Reader 1 were applied so they could be compared.

**Table 1:** Number of counts measured for the unirradiated samples for two different masses and three different grain sizes (data acquired with Reader 1).

**Stimulation type**	**Mass/g**	**Crumbled <0.1 cm**	**Grains **~**0.5 cm**	**Grains **~**1 cm**
λ = 890 nm	1	242 ± 8%	244 ± 7%	231 ± 20%
	2	240 ± 18%	261 ± 7%	257 ± 9%
λ = 470 nm	1	938 ± 8%	835 ± 5%	798 ± 4%
	2	896 ± 5%	843 ± 3%	869 ± 4%
Both	1	877 ± 7%	758 ± 13%	788 ± 10%
	2	846 ± 4%	861 ± 5%	823 ± 6%

**Table 2:** Number of counts measured for the samples irradiated with 1 Gy for two different masses and three different grain sizes (data acquired with Reader 1).

**Stimulation type**	**Mass/g**	**Crumbled <0.1 cm**	**Grains **~**0.5 cm**	**Grains **~**1 cm**
λ = 890 nm	1	414 ± 22%	503 ± 12%	705 ± 15%
	2	512 ± 9%	570 ± 14%	785 ± 21%
λ = 470 nm	1	9,713 ± 10%	6,821 ± 39%	13,719 ± 36%
	2	13,422 ± 10%	9,711 ± 14%	16,519 ± 21%
Both	1	9,421 ± 30%	7,298 ± 18%	11,496 ± 43%
	2	12,959 ± 37%	11,604 ± 27%	19,806 ± 25%

The justification for the different grain sizes of the crumbled crackers is related to the fact that salt may not be homogenously spread in the cracker. Moreover, two different masses of the samples were chosen to verify if this parameter would affect the results and, in case it does not, weighting of the samples may be omitted to save time.

There are a number of conclusions that can be drawn from Tables 1, 2. Firstly, the relative standard deviations of the measurements made on the unirradiated samples are on average smaller compared to the ones obtained from the measurements on the irradiated samples. This can be explained with the non-complete stabilization of the radiation-induced PSL signal intensity after 24 h from irradiation, as it will be shown later on (**Signal Stability**).

As far as different stimulations are concerned, the relative standard deviations from the average values are the highest when both, the blue and the infrared stimulations are applied simultaneously. To our knowledge, this kind of combined stimulation was never applied for salty crackers so, one can only speculate that the deviations in the case of such stimulation is a superposition of the standard deviations of each stimulation alone, but further investigation of this phenomenon is required. Moreover, blue light gave a better response compared to infrared light. This behavior is the consequence of the shorter wavelength of blue light directly influencing the depth of electromagnetic wave penetration into the material. Therefore, it has better accessibility to “shadowed” salt.

Still, the grain size is directly related to the non-homogeneous distribution of salt in the sample, independently on the stimulation wavelength. Therefore, the standard deviation of the response increases with the grain size for all applied light stimulations.

From presented results, it can be concluded that the grain size is a relevant parameter for the preparation of crackers as fortuitous samples. The samples with the smaller grain size are least affected by mass, while the samples with the largest grains (~1 cm) have bigger relative standard deviations, which is probably a consequence of the non-homogenous distribution of salt in the cracker.

### Dose Response

**Figure 1:**
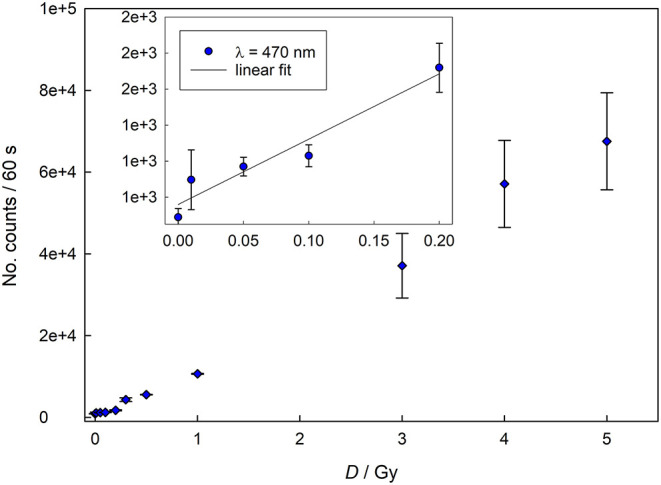
Dose response for blue light (470 nm) stimulation (data acquired with Reader 1).

**Figure 2:**
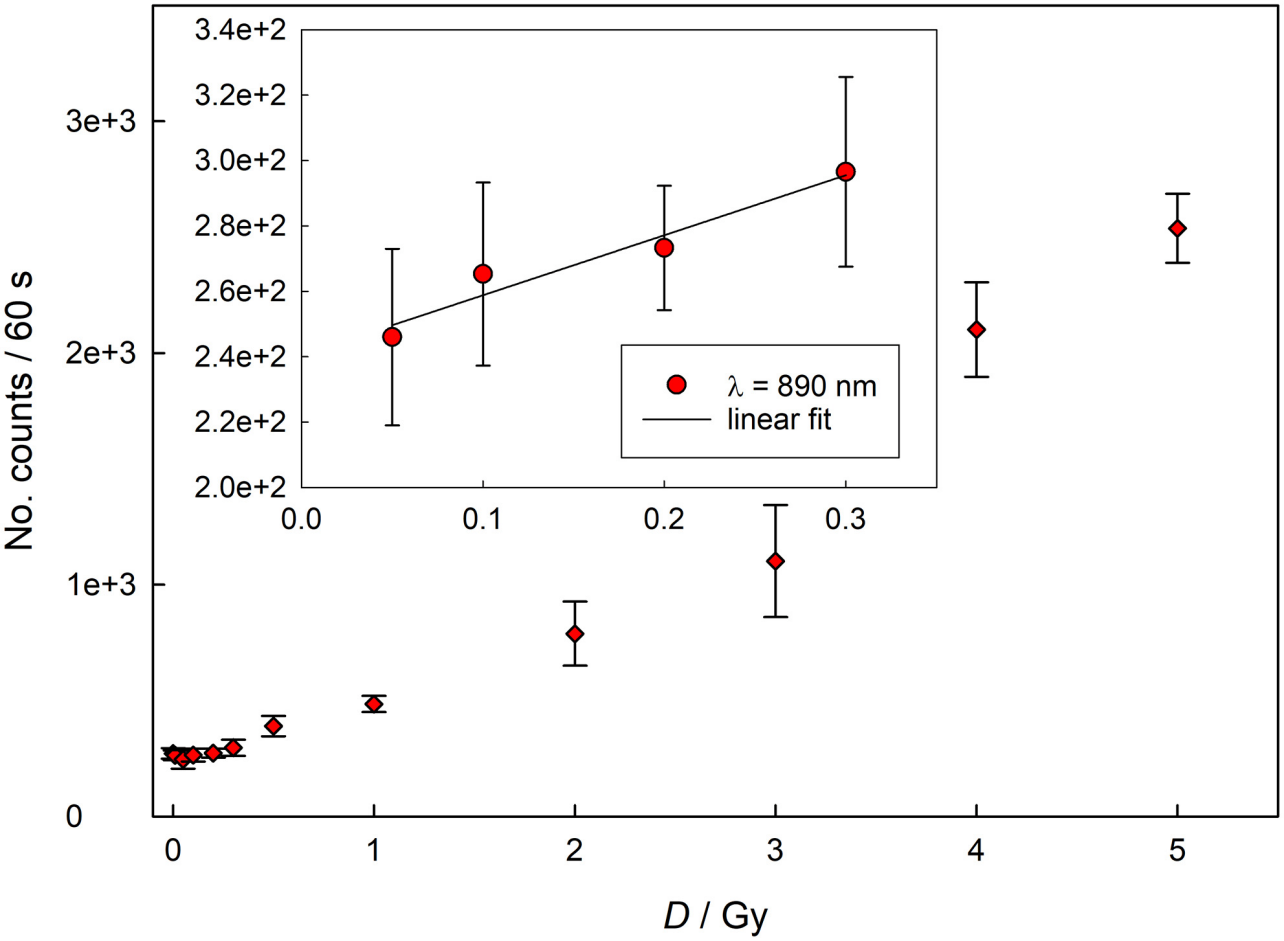
Dose response for infrared (890 nm) stimulation (data acquired with Reader 1).

Figures 1, 2 show the dose response, as well as the calibration curves, of completely crumbled crackers (<1 mm) determined for blue light and infrared stimulations using Reader 1.

**Figure 3:**
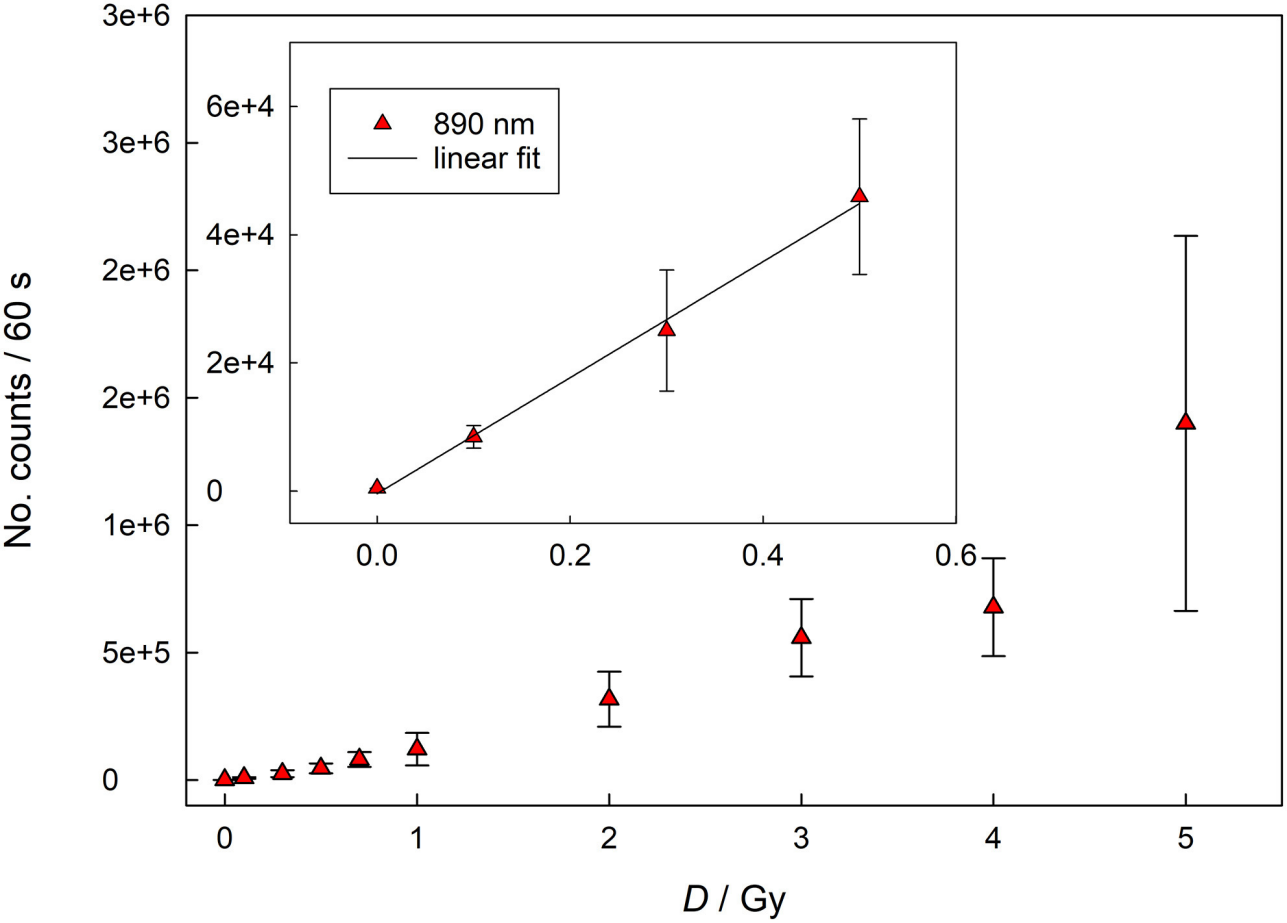
Dose response for infrared (890 nm) stimulation (data acquired with Reader 2).

In both cases the curves show a linear behavior for lower doses (up to 300 mGy for infrared stimulation and 200 mGy for blue light stimulation), while for higher doses the response assumes the characteristic supralinear dependence. Figure 3 represents dose response curve for data acquired with Reader 2. The dose response behavior appears similar to that one reported in Figure 2 with linear response up to 500 mGy, followed by typical supralinear trend. These findings are expected as a similar dose response behavior have been already reported for household salt and salty snacks (9, 12, 13).

The linear part of all curves was analyzed, and calibration curves were obtained by making a linear fit, where *k* is the slope and *l* is the *y*-intercept. The parameters of these curves and the respective standard deviations are shown in Table 3 and used for the calculation of the limit of detection. The results obtained for the *l* parameter (Table 3) are in agreement (within 1 SD) with the number of counts measured for the unirradiated samples (Table 1), which supports the reliability of fitting curves.

**Table 3:** Parameters obtained from the calibration curves for infrared (Reader 1 and Reader 2) and blue light stimulation (Reader 1).

	***k*/counts Gy^**−1**^**	***l*/counts**
λ = 890 nm (Reader 1)	183 ± 31	240 ± 6
λ = 470 nm (Reader 1)	3,628 ± 567	959 ± 58
λ = 890 nm (Reader 2)	90,529 ± 4,045	−389 ± 1,197

As there doesn't seem to be a standard method (14) for the calculation of the lowest measurable value that is statistically different from the zero-dose value, in this work two different approaches were taken. The first method for the calculation of the limit of detection is the one used by Currie et al. (15, 16). It consists in calculating the limit of detection from the average value of the zero-dose plus three corresponding standard deviations. The results of Reader 1 for the limits of detection for measurements are 70 mGy for the infrared stimulation and 20 mGy for the blue stimulation, while 100 mGy was obtained with Reader 2. The other method for the calculation of the limit of detection is the one reported by Geber-Bergstrand et al. (17). It considers the uncertainty of the calibration curves of the dose response. The limit of detection for both stimulations was calculated with the expression proposed in their study:

(1) $$\begin{array}{l} LOD=\;\frac{\langle S_{0}\rangle }{k}+3\cdot \left(\sqrt{{\left(\frac{\sigma_{S}}{\langle S_{0}\rangle }\right)}^{2}+{\left(\frac{\sigma_{k}}{k}\right)}^{2}}\cdot \frac{\langle S_{0}\rangle }{k}\right) \end{array}$$

where *LOD* is the limit of detection, 〈*S*_0_〉 is the average number of counts of the zero-dose samples, *k* is the slope of the calibration curve, σ_*S*_ is the standard deviation of 〈*S*_0_〉 and σ_*k*_ is the standard deviation of *k*. By inserting obtained results into the Equation 1, the limits of detection of Reader 1 are 0.37 Gy for the blue light stimulation and 2.33 Gy for the infrared stimulation and 0.6 Gy for Reader 2. The two methods give results that differ by an order of magnitude for both stimulations. Evidently, the standard deviations of the parameters of the calibration curves sensibly affected the obtained results. It has to be highlighted that the construction of statistical model is a delicate process as it is specific for each sample type and additional pertinent information about instrument.

**Figure 4:**
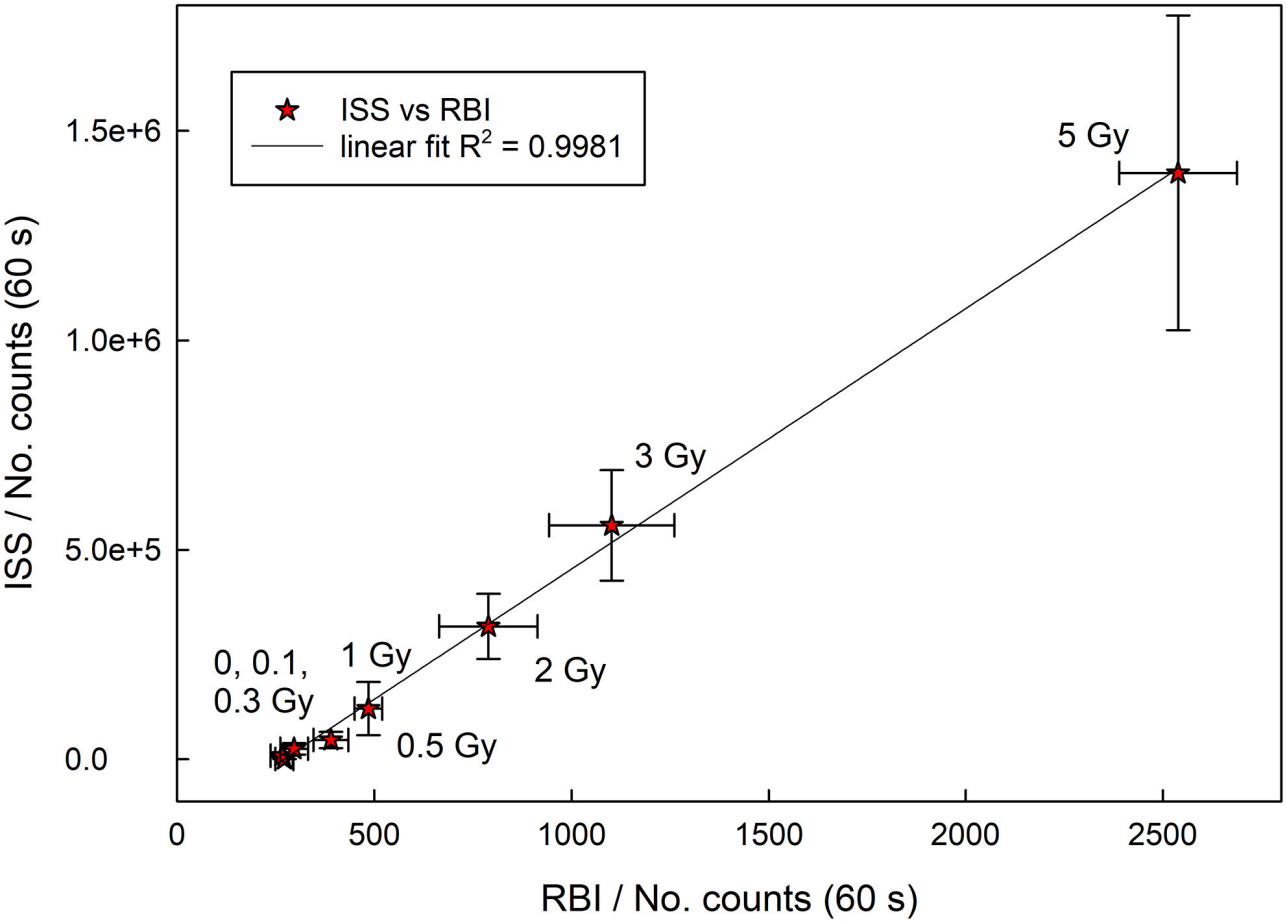
Total counts correlation between Reader 1 (RBI) and Reader 2 (ISS) for infrared (890 nm) stimulation.

Comparing the data reported for Reader 1 and Reader 2, it is evident that the PSL responses (total counts) recorded in the same conditions (infrared stimulation, same dose of ionizing radiation) with the two different models of PSL systems used in this study, are very different. The Reader 2 provides a more intense PSL responses with total counts which differ by more than one order of magnitude. Despite that, results obtained by two different Institutes and instruments can be compared and correlated as reported in Figure 4. Obtained results show that the proposed method can be successfully used for the triage according to the categorization protocol (1, 2). Furthermore, the results were not influenced by the difference in dose rates applied by the two Institutes.

This is an important result that suggests the possibility of harmonization of sample preparation and measurement procedure allowing different laboratories to simultaneously estimate the dose for this type of snacks and correlate results in the mass causality nuclear accident event when urgent dose assessment is required.

### Signal Stability

**Figure 5:**
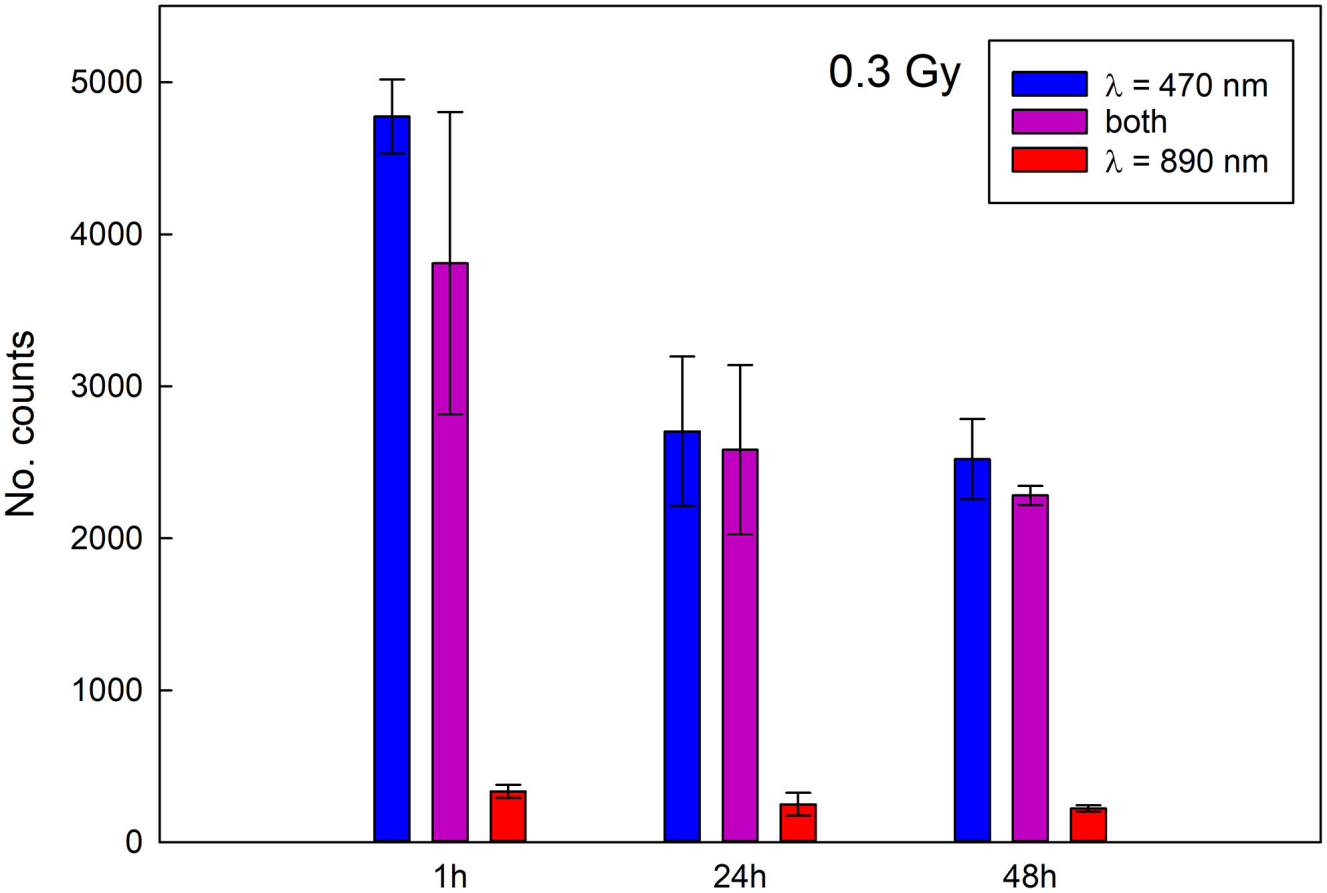
Signal stability for samples irradiated to 0.3 Gy (data acquired with Reader 1).

**Figure 6:**
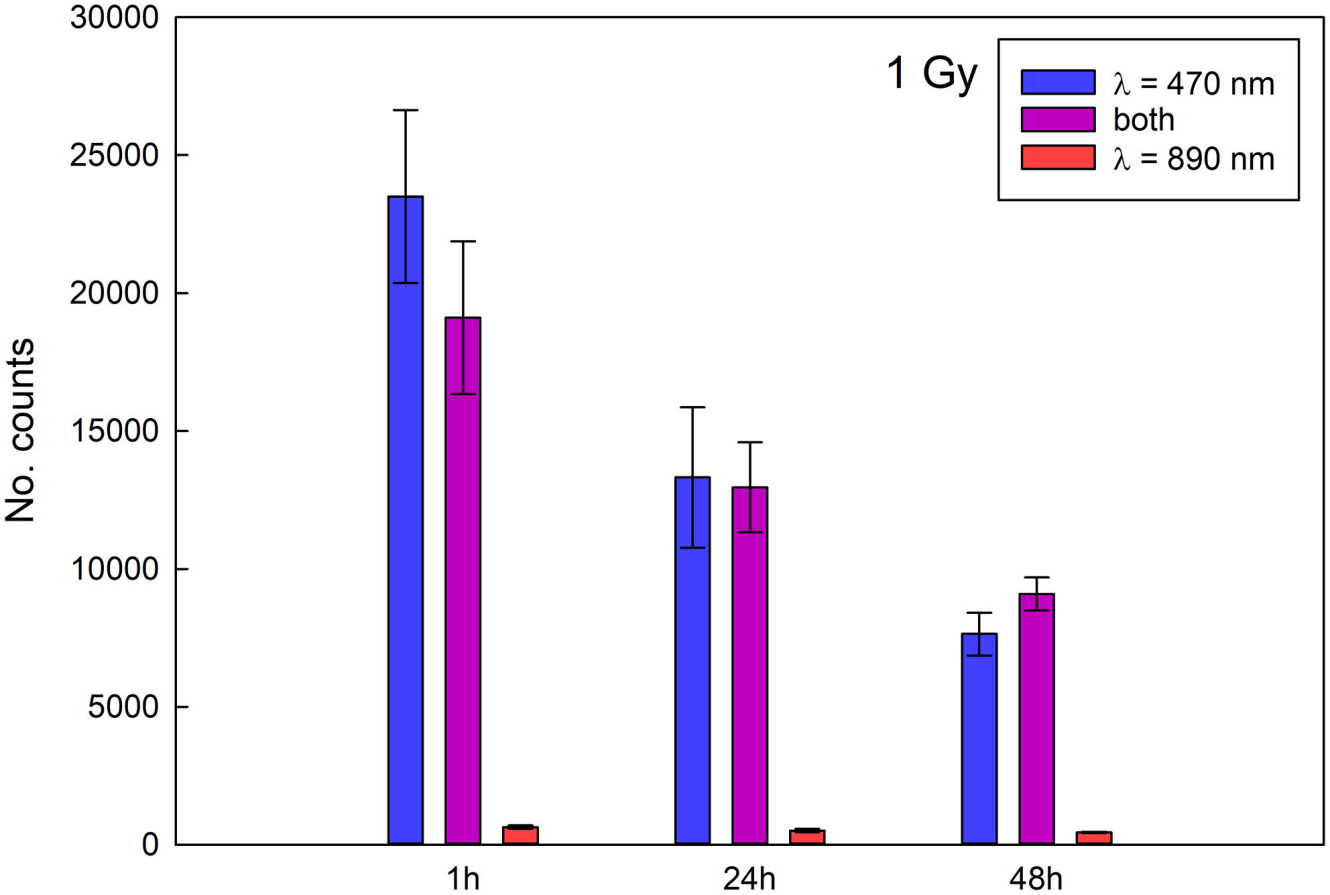
Signal stability for samples irradiated to 1 Gy (data acquired with Reader 1).

The stability of the signal was determined with Reader 1 for completely crumbled samples irradiated to 0.3 Gy and 1 Gy and for all three stimulations and is represented in Figures 5, 6. Blue light stimulation gives the strongest signal, followed by the combined stimulation, while the infrared stimulation gives the weakest signal. A decrease in the signal intensity is evident for both irradiation doses and for all the stimulations. During the first 24 h the signal obtained with blue light drops by about 40%, the one measured for combined stimulations by about 30% and about 20% for infrared stimulations for both irradiation doses. After 48 h the signal continues to weaken for the samples irradiated to 1 Gy, while it stabilizes for the sample irradiated with 0.3 Gy. The results are in line with fading properties obtained for pure NaCl (18) as expected as the main response of the crackers is due to the salt present in them.

On the other hand, the PSL signal recorded with Reader 2 on crackers irradiated with 0.3 and 3 Gy during the period from 48 h to 20 days after irradiation remains fairly constant. The number of counts for the sample irradiated with 0.3 Gy is on average 7,397 with a relative standard deviation of 22%, while for the cracker irradiated with 3 Gy this value is 67,582 with a relative standard deviation of 19%.

## Conclusion

In this work a specific brand of crackers (TUC original) was investigated with two PSL systems equipped with different stimulations (single or double). The main problems confronted in this paper were the dose response, the method of sample preparation, the advantages and disadvantages of the different stimulations and the signal stability in time.

The optimal sample preparation was determined by changing grain size, mass and stimulation. It was found that the results were the most accurate when the crackers were completely crumbled, had a larger mass and were exposed to blue light stimulation.

Although the instruments used in different Institutes have different response and limits of detection, the correlation is strong and opens the possibility of harmonization allowing simultaneous estimation of the dose for this type of snacks and the correlation of results in the mass causality nuclear accident event when urgent dose assessment is required.

In the case of salty snacks, the blue light stimulation is far more promising as it reduces the limit of detection.

The short (first 48 h) and long term (20 days) signal stability were determined for samples irradiated with 0.3 Gy and 3 Gy. The first one shows a fast decrease response for both doses, while the signal stays roughly stable 2 days after irradiation.

In general, the encouraging results as well as the simple sample preparation (crumbling of the cracker), rapidity of the analysis (60 s for each sample) and the simplicity of the operations make this method suitable for a rapid radiation triage of a large number of civilians in a mass casualty event with a portable PSL instrument. The transport of the instrument and the operator on the location of the event is probably the most time-consuming step in the estimation of the dose from the proposed samples. Until studies regarding that topic are not conducted, the advice is to protect the samples from conditions such as humidity (influence of water on the salt crystal), as well as keeping them away from UV light due to the possibility of bleaching (loss of recombination centers). The influence of other parameters, such as possible changes in environmental conditions, also remain open questions for future studies.

## Data Availability Statement

The raw data supporting the conclusions of this article will be made available by the authors, without undue reservation.

## Author Contributions

EB and NM-S: study concepts and design. MV, IE, MVK, EB, and MQ: data acquisition. MV and NM-S: data analysis and interpretation and statistical analysis. EB, MV, and NM-S: manuscript preparation. EB, MV, and NM-S: manuscript editing. EB, MV, NM-S, SD, and PF: manuscript review. All authors contributed to the article and approved the submitted version.

## Conflict of Interest

The authors declare that the research was conducted in the absence of any commercial or financial relationships that could be construed as a potential conflict of interest.
